# Epidemiological characteristics of injuries among elite adolescent flat-water kayak and canoe athletes

**DOI:** 10.3389/fpubh.2025.1608987

**Published:** 2025-09-01

**Authors:** Ke Gao, Yixin Deng, Xiao Zhou, Hongtao Zeng, Tomohiro Kimura, Shaoshuai Shen, Ziheng Pan, Na Han

**Affiliations:** ^1^College of Physical Education and Health, Guangxi Normal University, Guilin, China; ^2^School of Physical Education, Huazhong University of Science and Technology, Wuhan, China; ^3^Special Needs Education School for the Visually Impaired, University of Tsukuba, Tsukuba, Japan; ^4^School of Education and Welfare, Aichi Prefectural University, Nagakute, Japan; ^5^Graduate School of Comprehensive Human Sciences, University of Tsukuba, Tsukuba, Japan

**Keywords:** paddle sports, adolescent athletes, injury distribution, canoe, kayak

## Abstract

**Background:**

Sport injuries are now becoming a major issue affecting training in paddle sports. This study was to investigate the distribution and prevalence of injuries among adolescent flat-water kayak and canoe athletes.

**Methods:**

We performed a retrospective design study to survey one-hundred forty Chinese elite adolescent flat-water kayak and canoe athletes (89 kayakers and 51 canoers; 81 males and 59 females, with an average age of 16 years) using a self-reported questionnaire. The questionnaire investigated basic information and kayak- and canoe-related injuries over the past year. The primary outcome measures were the distribution of kayak- and canoe-related injuries and the injury rate per 1,000 training hours with a 95% confidence interval (CI).

**Results:**

A total of 207 injuries were reported from all the participants, including 138 injuries related to flat-water kayak and 69 injuries related to canoe. The most common injured site in flat-water kayak athletes was the lower back, followed by the shoulder, wrist, and knee. In flat-water canoe athletes, the most common injured site was the shoulder, followed by the lower back, back, and knee. Regarding injury rates, the flat-water kayak athletes showed 0.90 injuries per 1,000 training hours (95% CI = 0.75–1.05). The female kayak athletes had 1.02 injuries per 1,000 training hours (95% CI = 0.81–1.23), while the male kayak athletes had 0.74 injuries per 1,000 training hours (0.53–0.95). Notably, the injury rate per 1,000 training hours was significantly higher in female canoe athletes (1.51, 95% CI = 1.05 to 1.98) than in male canoe athletes (0.47, 95% CI = 0.30 to 0.64).

**Conclusion:**

The findings indicated that flat-water kayak- and canoe-related injuries mostly involved in the shoulder and lower back among Chinese elite adolescent athletes, with female athletes, particularly in canoe, being more susceptible to injury. These insights are critical for enhancing athlete injury prevention strategies and providing coaches and athletes with comprehensive reference data on injury.

## 1 Introduction

Flat-water kayak and canoe are internationally popular paddle sports in the summer that involve repetitive and specific motor paddle skills (1). Throughout the race, athletes begin from a stationary start and navigate a calm water course to the finish line (2–4). The techniques of paddle boarding sports (such as canoeing and kayaking) are similar, comprising of four phases of entry, pull, exit, and aerial (4, 5). Catch, immersion, extraction, and release are cyclic actions in canoeing, and in kayaking, although the upper extremities predominantly generate the force, all propulsive energy must still be transferred through the lower extremities to move the boat forward in the water (6).

Owing to the motor skill characteristics of paddle sports, injuries frequently occur. Improper paddling technique and repetitive muscle use can easily lead to overuse injuries. Combined with fatigue, these injuries can lead to imbalances in muscle power. Nearly half of all injuries occur on land, for example during training, scouting, or getting on and off the water. Still-water racers are more susceptible to overuse injuries due to prolonged repetitive motions (7, 8). Previous studies showed that the overuse injury rate was nearly double the traumatic injury rate (2.0 injuries per 1,000 h *vs.* 1.2 injuries per 1,000 h) (8). In kayak and canoe athletes aged 18–23 years, 48% of injuries associated with kayaking/canoeing were the upper limb injuries, and 34% were trunk injuries (3). A number of studies on kayak and canoe have shown that the injury rate was generally high, about 42.1–78.0%, with no significant difference between genders (male vs. female: 42.8–60.6% vs. 41.1–91.3%). Shoulder (27.0–35.6%) and lower back (12.0–27.0%) are two of the most common injured sites for kayak and canoe athletes (3, 9–11). In terms of the nature of injury, muscle (24.0%) and tendon injury (22.0%) were the most common types (3). The most common types of injuries occurred in the shoulder were acromioclavicular hypertrophy (55.0%), acromial spurs (40.0%), rotator cuff pathology (44.0%), and the common types of injuries occurred in the spine were vertebral bone fissure (17.5%), myofascial pain (15.9%), cervical spondylosis (12.7%), and intervertebral disc herniation (3.2%) (11).

All previous population-based epidemiological studies we found have grouped flat-water kayak and canoe athletes together. However, several differences in motor skills between the two sports existed that kayaking is bilateral and symmetrical, with a two-bladed paddle, whereas canoeing is unilateral and asymmetrical, with a single-bladed paddle. Pooling these factors obscures sport-specific injury factors due to their distinct biomechanical loading patterns, thereby preventing the development of precise, discipline-targeted prevention strategies. In addition, epidemiological studies on injuries in Chinese flat-water kayak and canoe athletes are scarce, especially among youth athletes. To improve the accuracy of injury prevention, enhance sports performance, and assure injury-free sports participation, this study aimed to identify the characteristics of injuries related to flat-water kayak and canoe among elite Chinese adolescent athletes.

## 2 Methods

Between June 25, 2023, and September 8, 2023, a retrospective study was conducted to collect records of kayaking and canoeing injuries from elite sports teams in areas where water sports are more popular in five provinces of China (Anhui, Hubei, Henan, Shaanxi and Sichuan). A total of 143 flat-water youth paddle athletes including 52 canoe athletes and 91 kayak athletes have been investigated. All of the athletes were at the provincial level or above, who have been trained in provincial teams and have participated in the national tournament. Injury surveillance was performed at water sports centers using a self-reported questionnaire modified from the previous studies (3). The self-reported questionnaire was used to collect the information related to demographic data (including gender, age, weight, height, dominant side, years of kayak/canoe training experience, daily training hours, weekly training days, and warm-up and cool-down time), and flat-water kayak and canoe related injury distribution over the past year. A total of 161 questions were designed to be divided into two main sections, past history and current medical history (12, 13). All the injuries were reported specifically regarding 14 anatomical sites including head, shoulder, arm, elbow, wrist, finger, back, lower back, hip, thigh, knee, lower leg, ankle, and foot. Coaches checked the questionnaire data at the beginning of the questionnaire distribution and at the final inclusion.

An injury was defined as any somatic discomfort occurring during training play that met one or more of the following three judgment criteria: (1) having to immediately discontinue the current canoeing and kayaking training; (2) being unable to participate in subsequent canoeing and kayaking training; and/or (3) needing medical care irrespective of the potential absence from training. Appropriate training volume is essential not only for minimizing injury risk but also for enhancing physical fitness (14). Accordingly, training volume of this study was defined as the duration of technical or physical training conducted under the supervision of a coach, excluding warm-up and cool-down periods.

The analysis of all data for the study commenced in 20 October 2023. The inclusion criteria of participants were as follows: (1) participants who practiced paddling with regular training in professional teams in China, (2) provided consent to participate in the study, and (3) completed the entire questionnaire. A total of 143 athletes were initially considered for the study. However, three athletes were excluded based on the following specific reasons: one athlete had missing information in the questionnaire, one had a history of surgery, and one had a current disease that could affect the study outcomes. Additionally, errors were minimized by excluding individuals who had not trained regularly.

This study was reviewed and approved by the Institutional Ethics Board of Tongji Medical College, Huazhong University of Science and Technology, China [Notification Number (2023) IEC (S172)]. This study followed the ethical principles of the Helsinki Declaration for human research. All participants and guardians of the minors were required to provide written informed consent prior to their participation in the study.

## 3 Statistical analysis

The Shapiro–Wilk test was used to check the normal distribution of the investigated data. The data of height presented normal distribution while age, weight, body mass index (BMI), years of training experiences, daily training hours, weekly training days, weekly training hours, training hours of a year presented non-normal distribution. Independent sample t-test and Mann–Whitney U test were used to compare the data between the flat-water kayak and canoe athletes, and the male and female athletes. χ^2^ test was used to analyze the comparisons of injury incidence between groups. Poisson distribution was applied to calculate the injury rate per 1,000 training-hours of exposure, with a 95% confidence interval (CI) also calculated for the injury rate per 1,000 training-hours of exposure. Statistical significance was considered as *p*-value of less than 0.05.

## 4 Results

A total of 140 participants including 89 flat-water kayakers and 51 flat-water canoeing athletes met the inclusion criteria. The mean age of the kayak athletes was 16.2 ± 2.5 years, with 45 (50.6%) male kayak athletes as well as 44 (49.4%) female kayak athletes; the mean age of the canoe athletes was 16.4 ± 2.6 years, with 36 (70.6%) males and 15 (29.4%) females. Among all the participants, 75 athletes (53.6%) including 40 males and 35 females, experienced at least one injury over the past year. In term of flat-water kayak and canoe themselves, 48 kayak athletes including 23 males and 25 females suffered from injuries while 27 canoe athletes including 17 males and 10 females suffered from injuries.

Table 1 shows the basic parameters of the flat-water kayak and canoe athletes. Significant differences in weight (kayak vs. canoe: 65.7 ± 10.1 kg vs. 71.1 ± 10.4 kg, *p* = 0.002) and daily training hours (kayak vs. canoe: 3.5 ± 1.4 h vs. 3.0 ± 1.2 h, *p* = 0.018) were found the between flat-water kayak and canoe athletes. Moreover, male canoe athletes showed significantly larger BMI (canoe vs. kayak: 22.8 ± 2.1 kg/m^2^ vs. 21.7 ± 3.3 kg/m^2^, *p* = 0.05) and longer training times weekly compared with male kayak athletes (canoe vs. kayak: 10.8 ± 2.4 vs. 8.6 ± 4.1, *p* = 0.047). In kayak athletes, significant differences in height (*p* < 0.001), weight (*p* < 0.001), training hours weekly (*p* = 0.009), and training hours a year (*p* = 0.009) existed between males and females. In flat-water canoe athletes, significant differences in height (*p* = 0.002) and weight (*p* < 0.001) existed between males and females. There were no significant differences in other variables.

**Table 1:** Basic parameters of the flat-water kayak and canoe athletes gender^a^.

Variable	Kayak		Canoe	
	Male (*n* = 45)	Female (*n* = 44)	Male (*n* = 36)	Female (*n* = 15)
Age, years	15.8 ± 2.2	16.6 ± 2.7	16.1 ± 2.5	16.9 ± 3
Height, cm	179.9 ± 7.5^‡‡^	169.8 ± 5.1	179.3 ± 4.6^‡‡^	168.6 ± 7.2
Weight, kg	70.4 ± 11.0^‡‡^	60.8 ± 6.0	73.5 ± 8.5^‡‡^	65.2 ± 12.6
BMI, kg/m^2^	21.7 ± 3.3^†^	21.1 ± 1.9	22.8 ± 2.1	22.9 ± 3.9
Years of experiences	2.5 ± 1.8	3.2 ± 3.1	2.1 ± 1.6	2.8 ± 2.9
Training hours, per day	3.3 ± 1.0	3.8 ± 1.7	3.0 ± 1.1	3.1 ± 1.3
Training sessions, per week	8.6 ± 4.1^†^	10.5 ± 3.2	10.8 ± 2.4	10.7 ± 2.6
Training hours, per week	27.6 ± 14.3^‡‡^	38.5 ± 20.6	32.9 ± 16.1	33.9 ± 17.8
Training hours, a year	1437.5 ± 742.8^‡‡^	2002.0 ± 1068.7	1709.5 ± 837.7	1770.7 ± 925.1

^a^Values are expressed as mean ± SD.

^‡^*p*-value < 0.05, between males and females; ^†^*p*-value < 0.05, between male kayak and canoe athletes; ^‡‡^*p*-value < 0.001, between males and females; **p-value < 0.001, between kayak and canoe.

**Figure 1:**
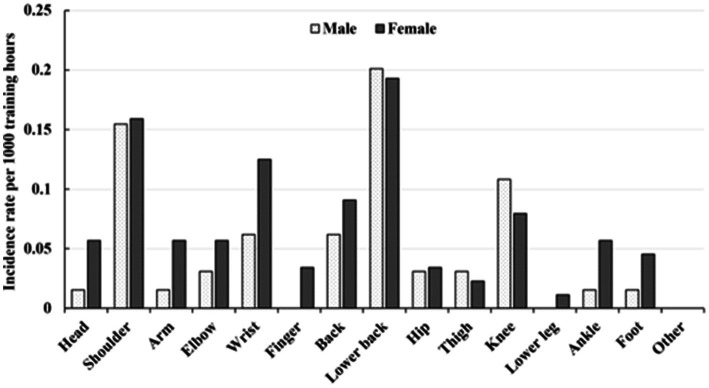
Injury rate per 1,000 training hours by anatomical site and sex in flat-water kayak athletes.

Injury rate per 1,000 training hours of anatomical sites in flat-water kayak athletes by sex is shown in Figure 1. Regardless of sex, the lower back injury showed the highest injury rate per 1,000 training-hours (male: 0.20, 95% CI = 0.10–0.31; female: 0.19, 95% CI = 0.10–0.28), followed by the shoulder (male: 0.15, 95% CI = 0.06–0.25; female: 0.16, 95% CI = 0.08–0.24). The knee injury rate per 1,000 training-hours (0.11, 95% CI = 0.03–0.19) ranked third among male kayak athletes while the wrist injury rate per 1,000 training-hours (0.12, 95% CI = 0.05–0.20) ranked third among female kayak athletes.

**Figure 2:**
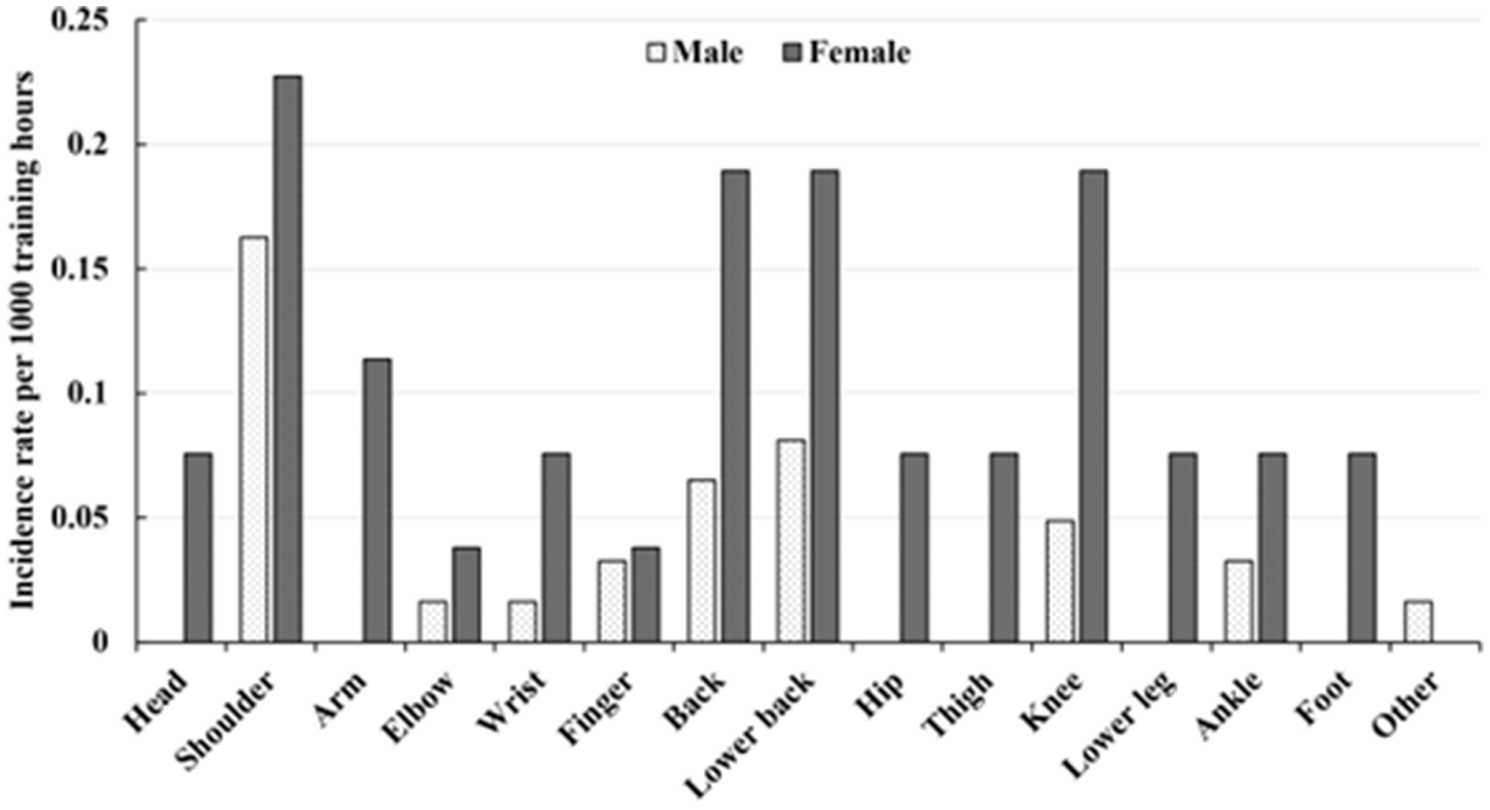
Injury rate per 1,000 training hours by anatomical site and sex in flat-water canoe athletes.

Injury rate per 1,000 training hours of anatomical sites in flat-water canoe athletes by sex is shown in Figure 1. Regardless of sex, the shoulder injury showed the highest injury rate per 1,000 training-hours (male: 0.16, 95% CI = 0.06–0.26; female: 0.23, 95% CI = 0.05–0.41). The lower back injury rate per 1,000 training-hours (0.08, 95% CI = 0.01–0.15) ranked second and the back injury rate per 1,000 training-hours (0.06, 95% CI = 0.00–0.13) ranked third among male canoe athletes while the lower back, the back, and the knee showed the same injury rate per 1,000 training-hours (0.19, 95% CI = 0.02–0.36) ranked second among female canoe athletes (Figure 2).

**Figure 3:**
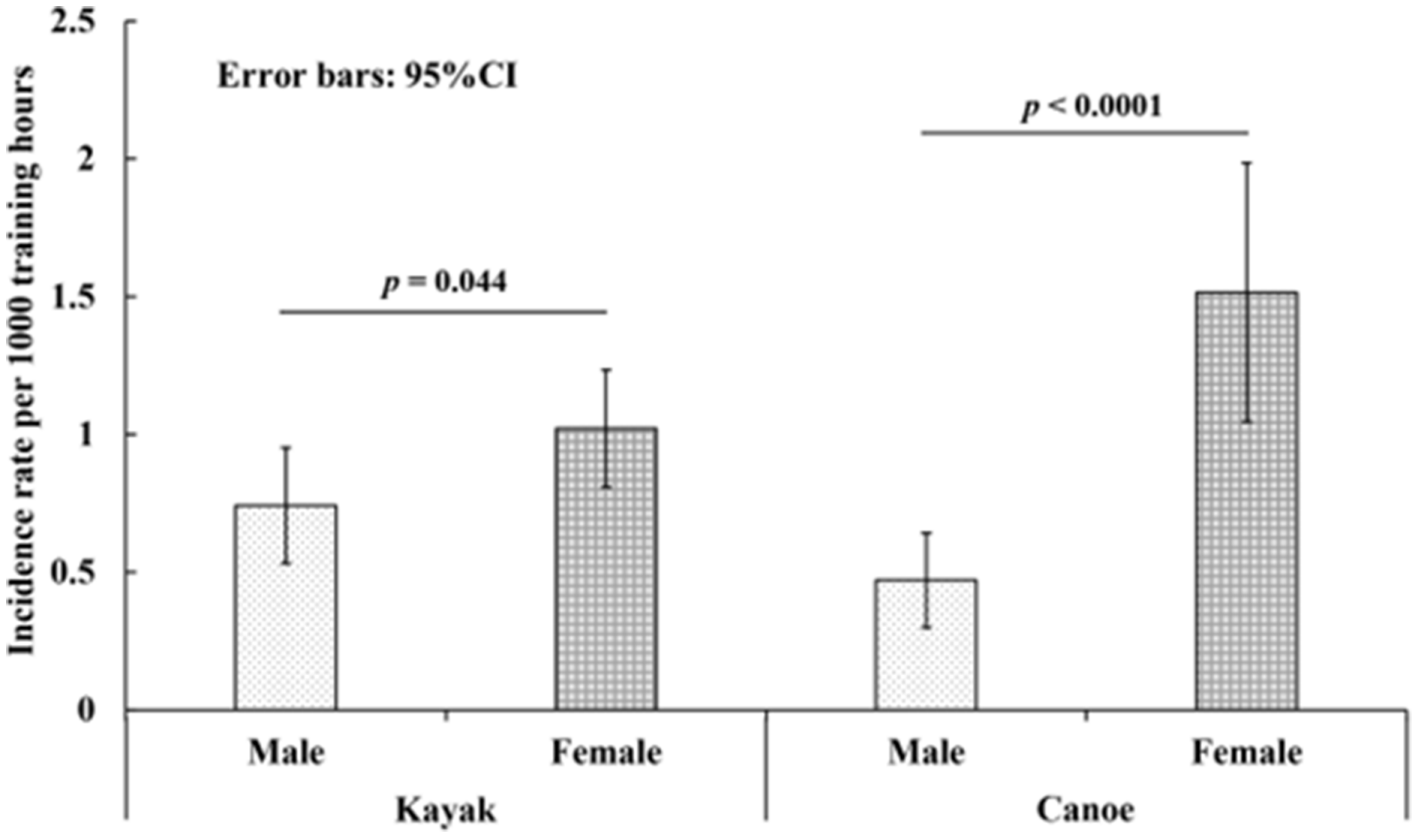
Injury rate per 1,000 training hours among flat-water kayak and canoe athletes by sex.

In terms of the injury rate per 1,000 training-hours of kayak, females showed 1.02 injures (95% CI = 0.81–1.23) per 1,000 training-hours, and males showed 0.74 injuries (0.53–0.95) per 1,000 training-hours. Regarding canoe, females showed significantly greater injury rate per 1,000 training-hours (1.51, 95% CI = 1.05–1.98) compared with males (0.47, 95% CI = 0.30–0.64) (Figure 3).

The distribution of injuries related to kayak in anatomical sites is shown in Table 2. Totally, 207 cases of injuries occurred, consisting of 138 cases of injuries related to kayak and 69 cases of injuries related to canoe. In kayak, the most common injury localized in the lower back, accounting for 21.7% of all the surveyed injuries, the shoulder ranked the second (17.4%), followed by the wrist (10.9%) and the knee (10.1%). Regardless of sex, the most common injury related to kayak localized in the lower back (male: 27.1%, female: 18.9%), followed by the shoulder (male: 20.8%, female: 15.6%). In the male athletes, the knee ranked third (14.6%) while the wrist ranked third (12.2%) in the female athletes. In canoe, the most common injury localized in the shoulder, accounting for 23.2% of all the surveyed injuries, the lower back ranked the second (14.5%), followed by the back (13.0%) and the knee (11.6%). Regardless of sex, the most common injury related to canoe localized in the shoulder (male: 34.5%, female: 15.0%). In the male athletes, the lower back ranked second (17.2%) while the back (12.5%), the lower back (12.5%), and the knee (12.5%) ranked second in the female athletes.

**Table 2:** Distribution of injury related to flat-water kayak and canoe athletes broken down by sex.

Body site	Kayak			Canoe		
	Male (*n* = 45)	Female (*n* = 44)	*p*-value	Male (*n* = 36)	Female (*n* = 15)	*p*-value
Head	1 (2.1%)	5 (5.6%)	0.086	0 (0.0%)	2 (5.0%)	-
Shoulder	10 (20.8%)	14 (15.6%)	0.308	10 (34.5%)	6 (15.0%)	0.391
Arm	1 (2.1%)	5 (5.6%)	0.086	0 (0.0%)	3 (7.5%)	-
Elbow	2 (4.2%)	5 (5.6%)	0.225	1 (3.4%)	1 (2.5%)	0.514
Wrist	4 (8.3%)	11 (12.2%)	**0.042**	1 (3.4%)	2 (5.0%)	0.144
Finger	0 (0.0%)	3 (3.3%)	-	2 (6.9%)	1 (2.5%)	0.878
Back	4 (8.3%)	8 (8.9%)	0.199	4 (13.8%)	5 (12.5%)	0.058
Lower back	13 (27.1%)	17 (18.9%)	0.639	5 (17.2%)	5 (12.5%)	0.111
Hip	2 (4.2%)	3 (3.3%)	0.627	0 (0.0%)	2 (5.0%)	-
Thigh	2 (4.2%)	2 (2.2%)	0.982	0 (0.0%)	2 (5.0%)	-
Knee	7 (14.6%)	7 (7.8%)	0.963	3 (10.3%)	5 (12.5%)	**0.025**
Lower leg	0 (0.0%)	1 (1.1%)	-	0 (0.0%)	2 (5.0%)	-
Ankle	1 (2.1%)	5 (5.6%)	0.086	2 (6.9%)	2 (5.0%)	0.347
Foot	1 (2.1%)	4 (4.4%)	0.472	0 (0.0%)	2 (5.0%)	-
Others	0 (0.0%)	0 (0.0%)	-	1 (3.4%)	0 (0.0%)	-
Total	48 (100%)	90 (100%)	**0.0002**	29 (100%)	40 (100%)	**0.0000**

The bold values indicate statistical significance, *p*-value <0.05.

## 5 Discussion

To the best of our knowledge, this might be the first to study and to compare the epidemiological characteristics of injuries among youth Chinese flat-water kayakers and canoeing paddlers. The findings of this study are: (1) over half of the flat-water kayak and canoe athletes experienced at least one injury; (2) regardless of flat-water kayak and canoe, injury frequently occurred in lower back and shoulder; (3) female athletes are susceptible to injury, especially female canoe athletes.

A previous study of elite Iranian dragon boat paddlers aged 21.6 (2.9)-22.0 (2.8) years reported that out of a total of 302 athletes (159 females, 143 males), 1,237 injuries were recorded (10.19 injuries/1,000 trainings or races), with females being more likely than males to have an injury to the upper limb (15). Regarding canoe, past studies on 122 elite flat-water canoe athletes (99 males, 23 females) with mean age 23.3 years at Spanish Water Kayaking Championship reported that 91.3% of females experienced injuries which was significantly greater than males of which 60.6% experienced injuries (9). In terms of injury rate per 1,000 sports-hours, previous study reported that six-hundred paddlers comprising of flat-water kayak and canoe athletes with mean age of 40.2 years showed 20.5 injuries per 1,000 h of marathon paddling during a 111-km paddling race (11). Another prospective-designed study showed that eight Brazilian females’ national canoe athletes with mean age of 19.5 years (a total of 87.5% of all the athletes experienced injuries) presented 5.06 injuries per 1,000 h of practice (16).

In this study, over half of the participants experienced at least one injury over the past year which is similar to the previous studies. The female flat-water canoe athletes showed significantly higher injury incidence compared with male canoe athletes, consistent with the previous study of canoe and with the 1.6 injuries per 1,000 h reported by Cassel et al. among 112 German adolescent canoe-sprint athletes, where females also showed higher risk for injury (8). Furthermore, the participants in the current study were adolescent athletes, a group experiencing significant growth-related changes, including rapid increases in body size and alterations in body composition. These changes are gender-specific; males typically exhibit substantial increases in muscle size and strength, enabling them to withstand greater loads and weights compared to females (17). Consequently, male athletes may experience less impact and fewer injuries from water pressure than female athletes. The physiological characteristics of the adolescent female athletes make them more susceptible to injury. Moreover, female athletes experienced significantly greater training hours a year (2002.0 h) compared to male athletes (1437.5 h). Under high loads of training, which may explain the higher injury rate observed in female athletes compared to male athletes in this study (11, 18). In addition, the flat-water kayak and canoe athletes presented similar injury rate per 1,000 training-hours was 0.09 injuries and 0.78 injuries, respectively which is significantly lower than the previous studies. The previous studies investigated injury happened during competition and training while our studies only investigated injury happened during training. The training is intense that might cause higher injury incidence.

In terms of anatomical sites, a past study on sprint kayak athletes reported that regardless of sex, shoulder was the most common injured site, followed by thoracic and lower back (3). Another previous study on paddle sports athletes including kayak athletes also reported that the top 3 injured anatomical sites were shoulder (31%), lower back (23.5%), and wrist (16.5%). In the current study, the top 3 injured sites were lower back, shoulder, and wrist among the kayak athletes which supports the previous studies (19). In addition, the only past study on kayak-related injury incidence per 1,000 h reported the injury rates of shoulder, lower back, and wrist were 27.2 injuries, 17.1 injuries, and 11.6 injuries per 1,000 h, respectively which is significantly greater than our studies. We suspect that the differences arose due to the design of the previous study, which investigated the injury over the past five years in participants aged 11–83 (19).

With regard to canoe, previous study reported that independent of sex, shoulder ranked the first (male: 38.2%, female: 52.9%) among 122 elite canoe athletes (99 males, 23 females) with mean age 23.3 years (9). Lower back injuries (18.2%) ranked the second among males while knee injuries (20.6%) among females. Lower back injuries (17.6%) ranked the third among female while knee injuries (13.6%) among males (9, 19). Another study showed that thoracic and thoracolumbar spine ranked the first, 35.9%, followed by lumbar and lumbosacral spine (20.51%), shoulder (17.95%), forearm (15.39%), and wrist (5.13%) among eight Brazilian females’ national canoe athletes (16). In our studies, the shoulder was the most frequent injured site in flat-water canoe athletes. Lower back injuries ranked the second among male athletes while back, lower back, and knee injuries ranked the second, which is in line with the previous studies.

The paddling cycle in both kayak and canoe can be divided into sequential phases: catch, power, and release (20, 21). Throughout this cycle, athletes repeatedly perform multi-joint movements—shoulder/trunk rotation, flexion/extension, and knee flexion/extension—to transfer energy from the lower limbs to the paddle (6, 21). Due to the repetitive and high-load nature of these actions, joint overload is common, leading to increased injury risk, particularly at the shoulder and lumbar spine (22, 23). Kayaking is a symmetrical, bilateral activity performed seated with a double-bladed paddle, whereas canoeing is asymmetrical and unilateral, performed kneeling with a single-bladed paddle (22, 23). In canoeing, the asymmetrical grip—one hand on the shaft and one on the handle—amplifies shoulder excursion and unilateral loading, which may explain the higher shoulder injury rates among canoeists (21, 22).

Although we have attempted to identify the epidemiological characteristics of sports injuries in flat-water kayak and canoe, several limitations should be acknowledged. Firstly, given that this study performed a retrospective survey design, recall bias is a limitation. Secondly, the surveyed data, lacking independent medical verification of the injuries, did not address the exact nature of the injuries in the different anatomical areas (e.g., fracture or strain), type (traumatic or overuse), and injury severity (minor, moderate, sever) or time to return to training. Thirdly, while our study included 140 participants, a larger sample size across a broader age range would provide more robust insights. Fourth, although the training hours have been investigated, we did not separately examine the time spent on on-water training and strength and conditioning training. Finally, the present study did not systematically examine intrinsic injury-predisposing factors—including, but not limited to, technical proficiency and physical fitness—in short- and long-distance flat-water kayak and canoe athletes. Therefore, the intrinsic factors of adolescent flat-water kayak and canoe should be the focus of future research.

## 6 Conclusion

The current study provides the epidemiological characteristics of flat-water kayak- and canoe-related injuries in Chinese adolescent athletes. Over half of the flat-water kayak and canoe athletes experienced at least one injury, mostly in the lower back and shoulder. Female athletes, particularly in canoe, were more susceptible to injury. These insights are critical for enhancing athlete injury prevention strategies and providing coaches and athletes with comprehensive reference data on injury.

## Data Availability

The raw data supporting the conclusions of this article will be made available by the authors, without undue reservation.
